# Supplementary material for: Seasonally migratory songbirds have different historic population size characteristics than resident relatives
Source: eLife. 2025 May 12;12:RP90848. doi: 10.7554/eLife.90848 (PMC12068868; doi:10.7554/eLife.90848)
Supplement: Supplementary file 1. [file elife-90848-supp1.docx]

| Table S1. Specimen data and NCBI-SRA numbers (PRJNA1112856). Vouchered specimens are housed in the following institutions: UAM (University of Alaska Museum), MSB (Museum of Southwestern Biology, University of New Mexico), LSUMNS (Louisiana State University Museum of Natural Science), and FMNH (Field Museum of Natural History). | | | | | | |
| --- | --- | --- | --- | --- | --- | --- |
| **Institution** | **Catalog #** | **Species** | **Year** | **Field No.** | **Locality** | **NCBI-SRA** |
| UAM | 28620 | *H. mustelina* | 2010 | KSW5403 | Belize: Toledo District; Big Falls | SRR29089747 |
| UAM | 27774 | *C. fuscescens* | 2007 | KSW5151 | Belize: Toledo District; Big Falls | SRR29089742 |
| UAM | 15202 | *C. guttatus* E | 1992 | KSW4013 | USA: Vermont; Brandon | SRR29089748 |
| UAM | 26337 | *C. guttatus* W | 2008 | UAMX5095 | USA: Alaska; Kodiak | SRR29089739 |
| UAM | 22642 | *C. minimus* | 2003 | KSW5000 | USA: Alaska; Fairbanks | SRR29089741 |
| n.a. (blood) | KF15K01 | *C. ustulatus swainsoni* | 2011 | KF15K01 | Canada: British Columbia, Kamloops | pending |
| n.a. (blood) | KF01K01 | *C. u. ustulatus* | 2011 | KF01K01 | Canada: British Columbia, Kamloops | SRS18060177 |
| UAM | 19996 | *C. bicknelli* | 2000 | KSW3633 | USA: Vermont; Mt Mansfield | SRR29089740 |
| UAM | 25341 | *C. aurantiirostris* | 2004 | MJM1154 | Panama: Chiriqui; El Salto | SRR29089749 |
| MSB | 31939 | *C. fuscater* | 2008 | MSB31939 | Peru: Amazonas; 4.5 km N Tullanya | SRR29089746 |
| UAM | 25098 | *C. frantzii* | 2004 | KSW4485 | Panama: Chiriqui; Volcan Baru | SRR29089744 |
| LSUMNS | 138784 | *C. gracilirostris* | 1990 | JMB1065; B-16270 | Costa Rica: San Jose; Cerro de la Muerte | SRR29089750 |
| UAM | 10352 | *C. mexicanus* | 1994 | PEP2489 | Mexico: Veracruz; Volcan San Martin | SRR29089743 |
| FMNH | 343305 | *C. occidentalis* | 1989 | MEX408 | Mexico: Oaxaca; Totontepec | SRR29089745 |

| Appendix 1-table 2. Data from PSMC analyses reflecting effective population sizes (*N_e_*) (X 10^4^ ) through history at depths > 50 Kyr (using variable generation times) and the five variables derived and analyzed from that output. Taxa shaded in gray are Neotropical residents. | | | | | |
| --- | --- | --- | --- | --- | --- |
| **Taxon** | **Mean *N_e_* (+ SD)** | **SD/mean** | **Degree of early growth 1 - (N_trough_/N_peak_)** | **Rate of early growth degree/deltaT** | **deltaT** |
| *Hylocichla mustelina* | 35.63 (+ 20.11) | 0.56 | 0.86 | 3.03E-07 | 2,834,197 |
| *Catharus fuscescens* | 95.77 (+ 56.07) | 0.58 | 0.83 | 1.79E-07 | 4,627,125 |
| *C. guttatus E* | 78.69 (+ 76.09) | 0.97 | 0.90 | 4.89E-07 | 1,850,522 |
| *C. guttatus W* | 40.15 (+ 24.11) | 0.60 | 0.81 | 3.59E-07 | 2,266,059 |
| *C. minimus* | 63.71 (+ 28.83) | 0.45 | 0.78 | 2.35E-07 | 3,323,013 |
| *C. ustulatus swainsoni* | 76.61 (+ 66.61) | 0.87 | 0.79 | 2.12E-07 | 3,740,764 |
| *C. ustulatus ustulatus* | 39.56 (+ 12.15) | 0.31 | 0.63 | 2.16E-07 | 2,909,628 |
| *C. bicknelli* | 46.90 (+ 24.12) | 0.51 | 0.78 | 3.01E-07 | 2,580,641 |
| *C. aurantiirostris* | 12.39 (+ 2.78) | 0.22 | -0.75 | -7.93E-07 | 942,318 |
| *C. fuscater* | 11.33 (+ 1.66) | 0.15 | 0.39 | 5.57E-07 | 713,817 |
| *C. frantzii* | 24.97 (+ 8.61) | 0.34 | 0.17 | 1.93E-07 | 886,294 |
| *C. gracilirostris* | 30.80 (+ 11.08) | 0.36 | 0.58 | 3.25E-07 | 1,775,757 |
| *C. mexicanus* | 37.10 (+ 16.18) | 0.44 | 0.75 | 2.92E-07 | 2,575,082 |
| *C. occidentalis* | 76.39 (+ 53.14) | 0.70 | 0.91 | 1.97E-07 | 4,595,855 |
| **Means (+ SD)** |  |  |  |  |  |
| migrants | 59.63 (+ 38.48)** | 0.61 (+ 0.21)* | 0.798 (+ 0.08)* | 2.87E-7 (+ 1.01E-7) | 3,016,494 (+ 875,860)* |
| residents | 32.16 (+ 15.58) | 0.36 (+ 0.19) | 0.342 (+ 0.59) | 1.284E-7 (+ 4.71E-7) | 1,914,854 (+ 1,489,246) |
| * *p* < 0.05; ** p < 0.01 |  |  |  |  |  |
